# Delayed endometrial decidualisation in polycystic ovary syndrome; the role of AR-MAGEA11

**DOI:** 10.1007/s00109-019-01809-6

**Published:** 2019-06-29

**Authors:** Kinza Younas, Marcos Quintela, Samantha Thomas, Jetzabel Garcia-Parra, Lauren Blake, Helen Whiteland, Adnan Bunkheila, Lewis W. Francis, Lavinia Margarit, Deyarina Gonzalez, R. Steven Conlan

**Affiliations:** 1 Reproductive Biology and Gynaecological Oncology, Swansea University Medical School, Singleton Park, Swansea, SA2 8PP UK; 2 Abertawe Bro Morgannwg University Health Board, Sketty Lane, Swansea, SA2 8QA UK

**Keywords:** PCOS, AR, MAGEA11, Decidualisation, Delay

## Abstract

**Abstract:**

Polycystic ovary syndrome (PCOS) is a common gynaecological disorder, with a prevalence of up to 12% of women of reproductive age, and is in part characterised by elevated circulating androgens and aberrant expression of androgen receptor (AR) in the endometrium. A high percentage of PCOS patients suffer from infertility, a condition that appears to be linked to mistimed and incomplete decidualisation critically affecting events surrounding embryo implantation. The aim of this study was to examine the involvement of MAGEA11, and the genome-wide role of AR in PCOS. We determined that elevated androgen levels on PCOS cells had an impact on the delayed and incomplete decidual transformation of endometrial cells. The AR co-regulator MAGEA11, a known enhancer of AR function, was constitutively overexpressed throughout the menstrual cycle of PCOS patients, co-localised in the nucleus of PCOS stromal tissue and cells and formed a molecular complex with AR. Genome-wide AR analysis in PCOS stromal cells revealed that AR targets included genes involved in cell death and apoptosis, as well as genes commonly dysregulated in endometrial cancer. Enhanced MAGEA11 and AR-mediated transcriptional regulation may impact on a correct endometrial decidualisation response, subsequently affecting endometrial receptivity in these infertile women.

**Key messages:**

- MAGEA11 and AR are overexpressed in hyperandrogenic PCOS patients.
- MAGEA11-AR overexpression in PCOS correlates with delayed decidualisation.
- AR and MAGEA11 associate in a molecular complex.
- AR directly regulates a unique set of genes controlling gene differentiation.

**Electronic supplementary material:**

The online version of this article (10.1007/s00109-019-01809-6) contains supplementary material, which is available to authorized users.

## Introduction

Polycystic ovary syndrome (PCOS) is a common gynaecological disorder affecting 4–12% of reproductive age women. Up to 44% of unexplained infertility cases display a PCOS morphology linked with ovulatory dysfunction and systemic hyperandrogenaemia. PCOS patients accumulate androgens due to defects in aromatase activity that prevents the conversion of androgens to oestrogens [1]. Despite ovulation, a proportion of PCOS patients remain infertile suggesting an important role for endometrial receptivity, orchestrated by embryo implantation and decidualisation, a phenomenon that involves dramatic morphological and functional differentiation of human endometrial stromal cells (hESCs) [2, 3]. In addition, endometrial cells from women treated with androgens exhibit altered growth and differentiation in vitro suggesting a potential decidualisation defect in PCOS patients [4].

Previously, we have shown that levels of apoptosis are reduced in stromal cells in both ovulatory PCOS and androgen-treated fertile samples [4]. This is accompanied by increased levels of anti-apoptotic factors Bcl2 and p27, and dramatic reductions in EGFR and WT1 expression in response to androgens [4]. Such alterations may contribute to the dysregulation of differentiation and cell cycle, thereby compromising the reproductive potential of PCOS patients [4].

The effects of androgens are mediated through androgen receptors (AR), nuclear hormone receptors that bind to and regulate the expression of specific target genes. AR functions through interactions with co-regulator proteins that result in the repression or activation of target gene transcription through modification of chromatin structure and regulation of RNA polymerase activity [5]. AR co-regulator proteins include MAGEA11, which has been shown to physically associate with the nuclear receptor in vitro [6, 7]. MAGEA family members are involved in the regulation of apoptosis, cell cycle progression, cellular differentiation, and proliferation [8–10]. Endometrial MAGEA11 expression has been shown in the secretory phase of the menstrual cycle in fertile women [11]. In vitro experiments demonstrated a progesterone receptor (PR) isoform B specific requirement for MAGEA11 in the activation of progesterone-regulated genes [7]. In prostate cancer, MAGEA11 is an androgen responsive gene and, following exposure of cells to androgens, co-localises to the nucleus with AR, where it functions as an AR co-regulator increasing AR transcriptional activity [12].

Here, we show that the addition of androgens disrupts the differentiation of endometrial stromal cells during the process of decidualisation in vitro. This process is accompanied by increases in the expression of MAGEA11, which is recapitulated in the endometrium of PCOS patients. Following treatment with dihydrotestosterone (DHT), both MAGEA11 and AR accumulate and co-localise in the nucleus both in vitro and in vivo in endometrial tissues obtained from PCOS patients. Genome-wide analysis of AR interactions using chromatin immunoprecipitation revealed that AR interacts with genes involved in the regulation of cell death and apoptosis including EGFR and WT1, supporting the notion that elevated androgens result in delayed decidualisation processes, and implicating AR and MAGEA11 in incorrect endometrial differentiation. This could impact on the critical timing process of embryo implantation, and thus human reproduction.

## Materials and methods

### Stromal cell isolation

Primary endometrial stromal cells (ESCs) were isolated from endometrial biopsies as previously described [4], yielding a ~ 95% pure stromal cell culture [13]. ESCs (6 × 10^5^ cells) were cultured in 6-well plates for 24 h prior to treatments. Confluent ESC monolayers were treated with cAMP (500 μM, A6885, Sigma, UK), DHT (10^−8^ M, A8380, Sigma), and DHT + cAMP (10^−8^ M + 500 μM) for 24 h, 48 h, and 72 h. RNA was extracted after treatments and cell morphology assessed throughout the duration of the experiment.

### Morphological analysis

Primary cells were washed in PBS and fixed for 15 min in 4% paraformaldehyde buffered in 0.1 M sodium cacodylate (pH 7.2). Standard light microscope images (Zeiss Axiovert™, UK) were captured and shape index was used to calculate changes in cell shape. ImageJ software was used to calculate the circularity of the cells and this was performed as previously described [14]. A perfect circle (indicating a decidualised cell) has a shape index of one while a straight line (indicating a non-decidualised stromal cell) has a shape index of zero.

### Enzyme-linked immune-sorbent assays

Human prolactin/IGFBP-1 DuoSet ELISA kits (DY682/DY871, R&D Systems, MN, USA) were used to analyse the presence of prolactin/IGFBP-1 in cell culture media. The target medium was collected from confluent monolayers of ESC cells treated for 48 h with cAMP and DHT. The protocols were performed as per manufacturer’s instructions and each sample was measured in triplicates.

### Patient samples

Patient recruitment and consent was carried out at the gynaecology clinics at Singleton Hospital, Swansea. Endometrial biopsies from women in a natural menstrual cycle were obtained for immunohistochemistry and in vitro studies. Patients had not received exogenous hormonal therapy at least two months before the procedure. Women with systemic diseases, sexually transmitted infections, or evidence of endometritis, endometrial hyperplasia, or endometrial polyp were excluded. The presence of chronic endometritis was ruled out by immunohistochemical analysis of biopsy samples. The phase of the natural menstrual cycle was confirmed by ultrasound and histological criteria; urinary luteinising hormone (LH) was used to document ovulation. The control group included women with proven fertility and regular menstrual cycles. The study group consisted of infertile women with PCOS; patients in control and study groups were matched with regard to body mass index (BMI) and smoking habits. PCOS was diagnosed based on the Rotterdam criteria of ultrasound and clinical and biochemical features of hyperandrogenism [15]. Anovulatory PCOS patients were oligomenorrhoeic or amenorrhoeic, and biochemical and clinical examinations confirmed the lack of ovulation. The ovulatory PCOS patients had confirmed polycystic ovaries on ultrasound, hyperandrogenism, and ovulated spontaneously with serum progesterone levels measured at LH + seven at least 30 nm/l, were nulliparous despite regular ovulatory cycles in the presence of patent tubes, and sperm parameters were normal. Blood samples were collected from patients at day two of the menstrual cycle for assessment of hormone levels using the electrochemiluminescence immunoassays (Elecsys® assays and Elecsys® 2010 immunoassay analyser, Roche) for progesterone, follicle stimulating hormone (FSH), luteinising hormone (LH), and testosterone, as well as radio-immunoassays (RIA, Beckman Coulter), to measure androstenedione. Endometrial biopsies were obtained by Pipelle endometrial sampling or by curettage concurrent to diagnostic laparoscopy; samples were divided in two groups for immunohistochemistry and *in vitro* studies.

A total of 116 patients were enrolled in the study, 51 of proven fertility and 65 infertile. Endometrial samples were obtained from 58 patients in the secretory phase [fertile (*n* = 33), infertile ovulatory PCOS (ovPCOS, *n* = 25)] and 37 patients in the proliferative phase. [fertile (*n* = 18), infertile ovPCOS (*n* = 19)]. Endometrial samples were also taken from 21 PCOS women who were anovulatory (anovPCOS). There were no statistically significant differences in the mean age or body mass index between the groups (Table 1). Both PCOS groups exhibited statistically higher levels of serum testosterone compared with the fertile group (Table 1).

**Table 1:** Patient demographics

	Fertile (*n* = 51)	Ovulatory PCOS (*n* = 44)	Anovulatory PCOS (*n* = 21)
Age	30 ± 4.6	29.0 ± 5.0 (*p* = 0.728)	29.1 ± 2.6 (*p* = 0.562)
Body mass index (BMI, kg/m^2^)	26.8 ± 4.9	29.1 ± 5.0 (*p* = 0.520)	32.3 ± 5.8 (*p* = 0.076)
Progesterone (ng/mL)	31 ± 5.28	27.45 ± 11.5 (*p* = 0.597)	N/A
FSH^1^ (mUI/mL)	7.4 ± 3.017	5.67 ± 1.37 (*p* = 0.135)	5.34 ± 2.57 (*p* = 0.2041)
LH^2^ (mUI/mL)	4.90 ± 2.221	12.70 ± 4.27 (*p* = 0.0004)	15.63 ± 4.94 (*p* = 0.0008)
Total testosterone (nmol/L)	0.76 ± 0.472	1.72 ± 0.313 (*p* = 0.1)	2.94 ± 0.108 (*p* = 0.001)
Free testosterone (nmol/L)	13.13 ± 6.12	18.5 ± 5.7 (*p* = 0.08)	37.15 ± 10.6 (*p* = 0.002)
Androstenedione (nmol/L)	3.43 ± 0.23	7.70 ± 1.6 (*p* = 0.006)	8.72 ± 1.1 (*p* = 0.0001)

^1^*FSH* follicle stimulating hormone

^2^*LH* luteinising hormone

### Immunohistochemistry

Preparation of formalin-fixed paraffin-embedded (FFPE) samples, nuclei-staining, and immunohistochemistry was performed as previously described [16]. Positive (tonsil) and negative (endometrium) tissue sections were used as controls. Rabbit polyclonal antibodies for anti-MAGEA11 (T1241, Epitomics®, California, USA) and for anti-AR (N-20, Santa Cruz®, California, USA) were used (1/50). Slides were evaluated using the H-scoring system by three independent scorers blinded to patient diagnosis, demographics, and timing of biopsy [16].

### RNA isolation and qRT-PCR

RNA isolation, quantification, and qRT-PCR analysis using gene-specific primer-pairs (available on request) were performed as previously described [4]. Expression levels were normalised to an internal reference gene (*GAPDH*). Expression was calculated as a ratio between treated and control samples for each gene. Densitometry analyses were performed using Image Lab software (BioRad, UK).

### Immunofluorescence confocal microscopy

ESCs (2 × 10^5^ cells) were grown to 80% confluence on 8-well chambered borosilicate coverglass (Thermo Scientific Ltd., UK). At 24 h post induction of decidualisation (+DHT), cells were fixed with 4% paraformaldehyde, permeabilised with 0.1% Triton X-100 (Sigma), and blocked with 3% BSA in PBS for 1 h. Primary antibodies, anti-AR (ab9474, Abcam®, Massachusetts, USA), and anti-MAGEA11 (ab60043, Abcam®) diluted in 3% BSA PBS were incubated overnight at 4 °C, followed by 1 h incubation with secondary antibodies, Alexa488 (green, mouse anti-AR) and Alexa TexasRed (red, rabbit anti-MAGEA11). Cells were washed with PBS extensively between all steps, counterstained before imaging with NucBlue reagent (Life Technologies) and imaged using an LSM710 confocal fluorescence microscopy system (Zeiss, UK).

### Protein blotting and co-immunoprecipitation

Protein blotting was performed as previously described [14]. For co-immunoprecipitation, PCOS stromal cells were grown to confluency and subsequently exposed to 0.1 μM DHT for 24 h before cells were fixed with 4% paraformaldehyde in order to stabilise protein complexes. Total protein was extracted using RIPA buffer and pre-cleared by rotation with A/G PLUS-Agarose beads (sc-2003, Santa Cruz). The lysate was subjected to anti-AR antibody (ab9474, mouse monoclonal) O/N incubation (4 °C), followed by another incubation with A/G PLUS-Agarose beads (2 h). After several washing steps, the samples were separated by SDS-PAGE and probed using an anti-MAGEA11 antibody (T1241, rabbit polyclonal).

### Chromatin immunoprecipitation and DNA sequencing

PCOS ESCs (1.2 × 10^6^ cells) were cultured in 75-cm^2^ flasks for 24 h prior to DHT treatment (48 h). DHT-treated PCOS ESC cells were then fixed using 1% formaldehyde solution (Sigma®), quenched with 2.5 M Glycine (Sigma®), and centrifuged following Active Motif’s Epigenetic Services ChIP Cell Fixation Protocol instructions. The resulting pellet was sent to Active Motif® for sequencing, alongside with an anti-AR antibody (ab9474), which was used to probe for AR-target region enrichment. Raw ChIP-seq data is deposited in the GEO Dataset, with accession number GSE119432.

### MEME-Suite

FASTA data files obtained from the AR ChIP-seq performed on PCOS tissue were loaded onto the MEME-Suite. First, the sequence analysis tool DREME (Bailey 2011) was used to discover relatively enriched motifs, comparing the AR ChIP-seq sequences with control shuffle sequences. Next, the motif comparison tool Tomtom (Gupta et al. 2007) was used to compare the DREME-enriched motifs against a database of known motifs, providing a list of matches and their correspondent *p* values.

### Analysis of the ChIP-seq data and Venn diagram

Alongside with the correspondent FASTA files, Active Motif® provided a list of 2798 genes. In most cases, the gene sequence encompassed the associated ChIP-seq peak location, although some upstream or downstream peaks were linked to gene targets through the identification of nearby promoters. Each gene was associated with a peak value, an average of peak values in case the gene contains several peaks, which were filtered to obtain a list of 577 high-intensity peaks (peak value > 20). The list of 577 high-intensity peaks was compared with a list of AR binding sites in human prostate cancer tissues (Sharma et al. 2013) using a Venn diagram (Hulsen et al. 2008).

### Genomic Regions Enrichment of Annotations Tool

The filtered genomic localisation of 6296 AR-PCOS peaks (peak value >20) was loaded onto Genomic Regions Enrichment of Annotations Tool (GREAT) (McLean et al. 2010) in order to analyse the functional significance of ChIP-seq-identified cis-regulatory regions (gene ontology).

### Statistical analyses

Data distributions were assessed for normality using the Kolmogorov Smirnov test. Non-normally distributed data was analysed with the Mann-Whitney *U* test applied in a post hoc to determine statistical significance. For normally distributed data, an ANOVA test followed by a *t* test was used to determine significant differences between groups. The test statistic and corresponding *p* value were reported. All data analyses were performed using SPSS version 13.0 (SPSS, Illinois, USA).

## Results

### Mesenchymal to epithelial transition is blocked in PCOS stromal cells

**Fig. 1:**
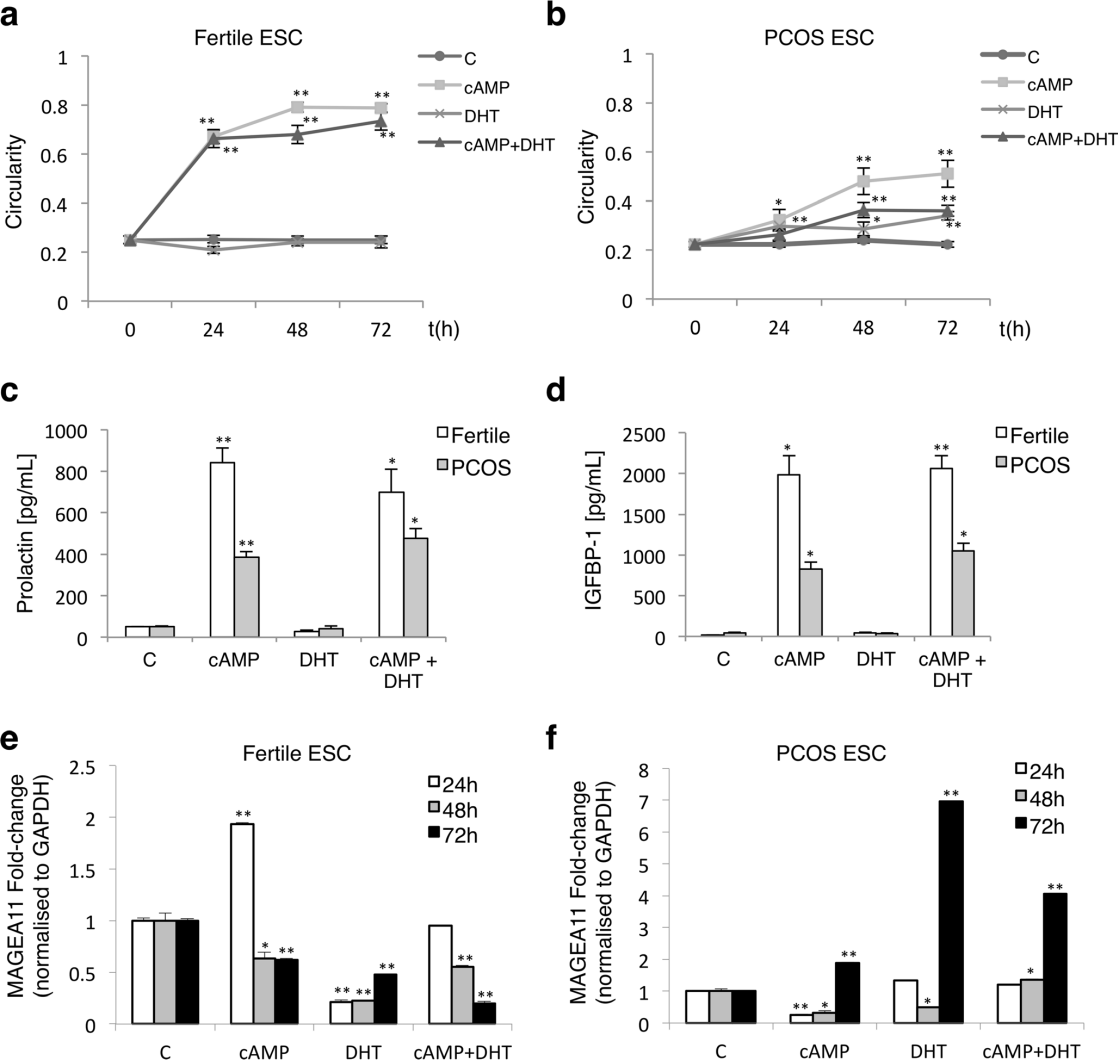
Regulation of MAGEA11 expression in ESC during in vitro decidualisation. Endometrial stromal cells (ESC) isolated from biopsies of fertile (*n* = 8) and PCOS (*n* = 9) patients were left untreated (C), incubated with cyclic AMP (cAMP, 0.5 mM), dihydrotestosterone (DHT, 10^−6^ M) or a combination of both for 24 to 72 h. ESCs were obtained from cells in the secretory phase of the cycle. Cell morphology was recorded throughout the experiment to document decidual changes. **a**, **b** ESC shape changes were assessed as described in the “Materials and Methods” section. ImageJ software was used to calculate the circularity of the cells (*n* = 20/group), with a perfect circle having a value of one, while a straight line has a circularity value of zero. **c**, **d** Cell media was extracted after ESCs were isolated and treated for 48 h. ELISA experiments were carried out on the conditioned media to establish the concentration of key proteins (prolactin/IGFBP-1) at the secreted level. Values were compared with their corresponding untreated cells. **e**, **f** After incubation, cell pellets were collected and RNA extracted to analyse MAGEA11 transcript levels by qRT-PCR. All values represent the average and standard deviation. Statistical analysis of the data was performed between samples of the same clinical sub-group using an ANOVA test followed by a *t* test (treated vs untreated). *p ≤ 0.05 and **p ≤ 0.01 were considered significant

Decidual transformation involves a mesenchymal to epithelial (MET)-like phenotypic transition in hESC in response to cyclic AMP (cAMP) in a normal menstrual cycle [17]. We assessed hESC isolated from primary tissue samples for a decidual response by calculating the cell shape index for MET. Cell shape index is a dimensionless quantitative measure of cell morphology acquired from standard light microscope images [18]. Following an initial exposure to cAMP to trigger differentiation, stromal cells underwent a rapid MET transition, morphologically transitioning from elongated to rounded cells with the calculated shape index approaching one (Fig. 1a and Supplementary Fig. 1). The MET-like response was initiated within the first 24 h following cAMP treatment and was sustained over the duration of the experiment, demonstrating that cAMP could effectively stimulate the transition. This observed MET-like effect was not impaired when fertile cells were simultaneously treated with cAMP and DHT, demonstrating that MET in fertile cells is unaffected by DHT. In contrast, cells derived from PCOS biopsies exhibited a delayed and only partial shape transition (Fig. 1b). After 24 h the shape index was 0.3, and increased slowly up to a maximum of 0.5 after 72 h in response to cAMP. When cAMP and DHT were used in combination in PCOS cells, the extent of the MET-like response remained partial, with a shape index of 0.3 at each time points (Fig. 1b).

Prolactin and IGFBP-1 are known indicators of MET in the context of hESC [17]; accordingly, the expression of these markers was quantified using enzyme-linked immune-sorbent assays (ELISA) (Fig. 1c, d). In fertile cells, prolactin and IGFBP-1 levels were significantly increased following exposure to cAMP (prolactin: *p* < 0.01; IGFBP-1: *p* = 0.025) and cAMP + DHT (prolactin: *p* = 0.03; IGFBP-1: *p* < 0.01). In accordance with the morphological response observed in PCOS patients (Fig. 1b), PCOS cells exhibited a limited upregulation of prolactin and IGFBP-1 following exposure to cAMP (prolactin: *p* < 0.01; IGFBP-1: *p* = 0.035) and cAMP + DHT (prolactin: *p* = 0.03; IGFBP-1: *p* = 0.02). These results are consistent with previous observations and indicate that the decidual response is either delayed or inhibited in PCOS patients.

### In vitro response to DHT and cAMP treatments

Steroids (DHT) and second messengers (cAMP) regulate the expression of the AR co-regulator MAGEA11 in human endometrial cell lines [11]. Accordingly, treatment of hESCs isolated from fertile samples with cAMP triggered a rapid and short-lived induction of MAGEA11 expression after 24 h (fold change = 1.9, *p* < 0.01), which decreased after 48 h and 72 h (Fig. 1e). For PCOS cells, the opposite response was observed, with MAGEA11 upregulation delayed until 72 h (fold change = 1.88; *p* < 0.01) (Fig. 1f). This delayed expression of MAGEA11 is consistent with previous observations, as the timing of maximal MAGEA11 mRNA expression in fertile women coincides with the window of receptivity to embryo implantation in the mid-secretory phase of the menstrual cycle [11], and thus suggests an anomalous decidual response in PCOS patients.

Cells were then treated with DHT, and in fertile hESC, MAGEA11 expression was significantly reduced at all time points (24 h: fold change = 0.23, *p* < 0.01; 48 h: fold change = 0.24, *p* < 0.01; 72 h: fold change = 0.48, *p* < 0.01), and when used in combination with cAMP, was further reduced after 48 and 72 h (48 h: fold change = 0.54, *p* < 0.01; 72 h: fold change = 0.21, *p* < 0.01) (Fig. 1e). This suggests that DHT through the action of AR, which is expressed at low levels in the mid-secretory phase of the menstrual cycle [11], represses normal MAGEA11 expression. In contrast, in PCOS samples, there was a dramatic increase in MAGEA11 expression 72 h post-treatment (DHT: fold change = 7.03, *p* = 0.01; cAMP + DHT: fold change = 4.05, *p* = 0.03, Fig. 1f) indicating that MAGEA11 is positively regulated by DHT in PCOS stromal cells.

### Expression of MAGEA11 and AR in proliferative and secretory phase endometrial biopsies

To determine whether the expression of MAGEA11 in vivo was similar to that observed in vitro, biopsies obtained from proliferative endometrium of fertile and PCOS patients were assessed using immunohistochemistry (IHC).

**Fig. 2:**
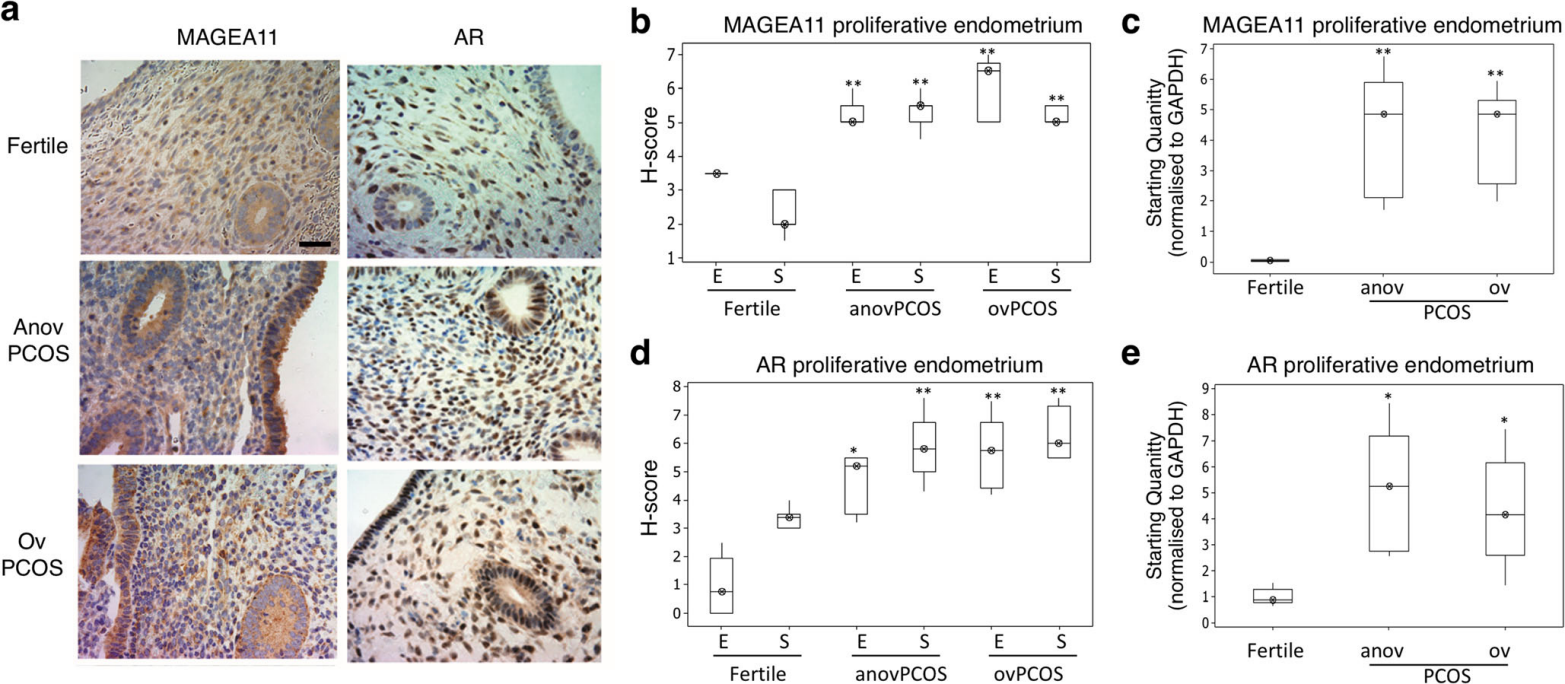
MAGEA11 expression in proliferative phase endometrium. Proliferative phase endometrium from fertile, anovulatory PCOS and ovulatory PCOS patients was analysed for the expression of MAGEA11/AR as described in the “Materials and Methods” section. **a** Immunohistochemistry (IHC) figures showing MAGEA11/AR expression; IHC images display × 10 magnification. Scale = 50 μm. **b**, **d** Analysis of the IHC scores (H-score) for MAGEA11 and AR antibodies. Fertile (*n* = 18); anovulatory PCOS (anovPCOS, *n* = 21); ovulatory PCOS (ovPCOS, *n* = 25); E, epithelial; S, stromal. **c**, **e** RNA samples obtained from endometrial biopsies were analysed for the expression of MAGEA11/AR transcript levels as described in the “Materials and Methods” section. Fertile (*n* = 10); anovPCOS [12]; ovPCOS (*n* = 6). All values represent median and inter-quartile ranges (box and whisker). Statistical analyses were performed using the Mann-Whitney *U* test. *p ≤ 0.05 and **p ≤ 0.01 were considered significant

**Fig. 3:**
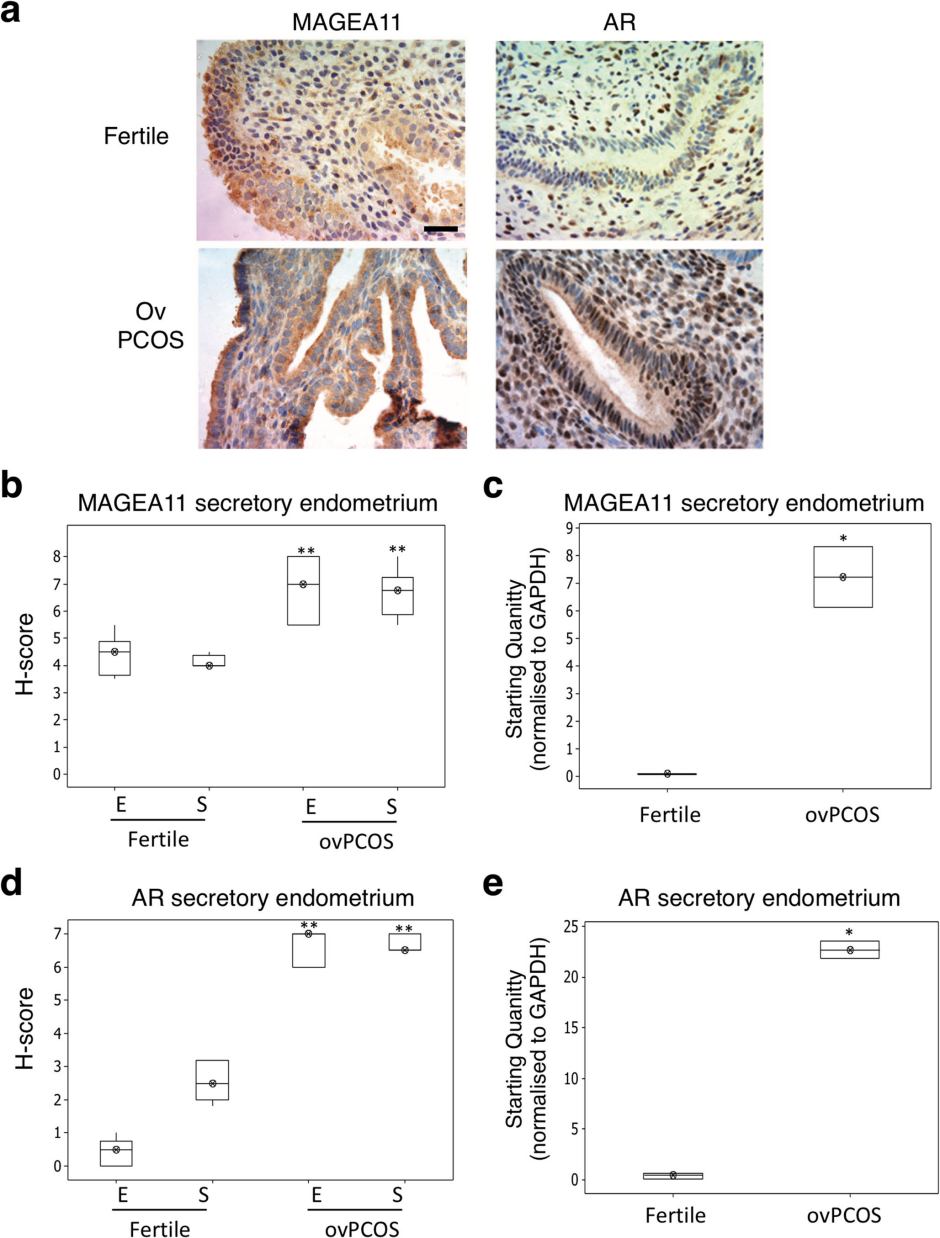
MAGEA11 expression in secretory phase endometrium. Secretory phase endometrium from fertile (*n* = 33) and ovulatory PCOS (ovPCOS, *n* = 19) patients was analysed for the expression of MAGEA11/AR as described in the “Materials and Methods” section. **a** Inmunohistochemistry (IHC) figures showing MAGEA11/AR expression; IHC images display × 10 magnification. Scale = 50 μm. **b**, **d** Analysis of the IHC scores (H-score) for MAGEA11 and AR antibodies. Fertile (*n* = 33); ovPCOS (*n* = 19); E, epithelial; S, stromal. **c**, **e** RNA samples obtained from endometrial biopsies were analysed for the expression of MAGEA11/AR transcript levels as described in the “Materials and Methods” section. Fertile (*n* = 12); ovPCOS (*n* = 8). All values represent the median and inter-quartile range (box and whisker). Statistical analysis was performed using the Mann-Whitney *U* test. *p ≤ 0.05 and **p ≤ 0.01 are considered significant

MAGEA11 was present in epithelial and stromal compartments of fertile endometrium, with expression restricted to the cytoplasm (Fig. 2a, b and Supplementary Fig. 2), and only low levels of MAGEA11 mRNA were detected in whole tissue extracts (Fig. 2c). Secretory phase expression was significantly higher in epithelial (*p* = 0.03) and stromal tissue (*p* < 0.01) compared with proliferative phase samples in fertile samples (Fig. 3a, b and Supplementary Fig. 2), confirming that MAGE11 expression is increased around the window of implantation [11]. Similarly, MAGEA11 mRNA expression showed an almost 3-fold increase in the secretory phase compared the proliferative phase in whole tissue samples (Fig. 3c, *p* = 0.01).

In ovulatory (ovPCOS) and anovulatory PCOS (anovPCOS), MAGEA11 levels were significantly higher than in fertile patients (Figs. 2 and 3 and Supplementary Fig. 2). Proliferative phase ovPCOS epithelial (*p* < 0.01) and stromal (*p* < 0.01) compartments showed high levels of expression, which were further increased in the secretory phase (epithelial and stromal *p* < 0.01). Similarly, MAGEA11 levels were significantly elevated in anovPCOS samples in both the epithelial (*p* < 0.01) and stromal (*p* < 0.01) compartments (Fig. 2a, b and Supplementary Fig. 2). MAGEA11 mRNA expression levels were significantly elevated in both ovPCOS (proliferative, *p* = 0.01; secretory *p* = 0.05) and anovPCOS (*p* = 0.0011) compared with fertile sample levels (Figs. 2c and 3c).

AR staining in fertile endometrium was higher during the proliferative phase of the cycle and predominantly expressed within the stromal compartment (Figs. 2d and 3d). Consistent with other studies [19, 20], this suggests that in fertile endometrium, an increase in MAGEA11 is unlikely to be due to AR activity, as the two molecules are expressed in different phases of the menstrual cycle. In PCOS samples, AR staining (Figs. 2d and 3d) was higher compared with the fertile samples, and MAGEA11 and AR expression occurred concurrently throughout the cycle (Figs. 2e and 3e).

### MAGEA11 co-localises with AR in the nuclei of PCOS stromal cells

**Fig. 4:**
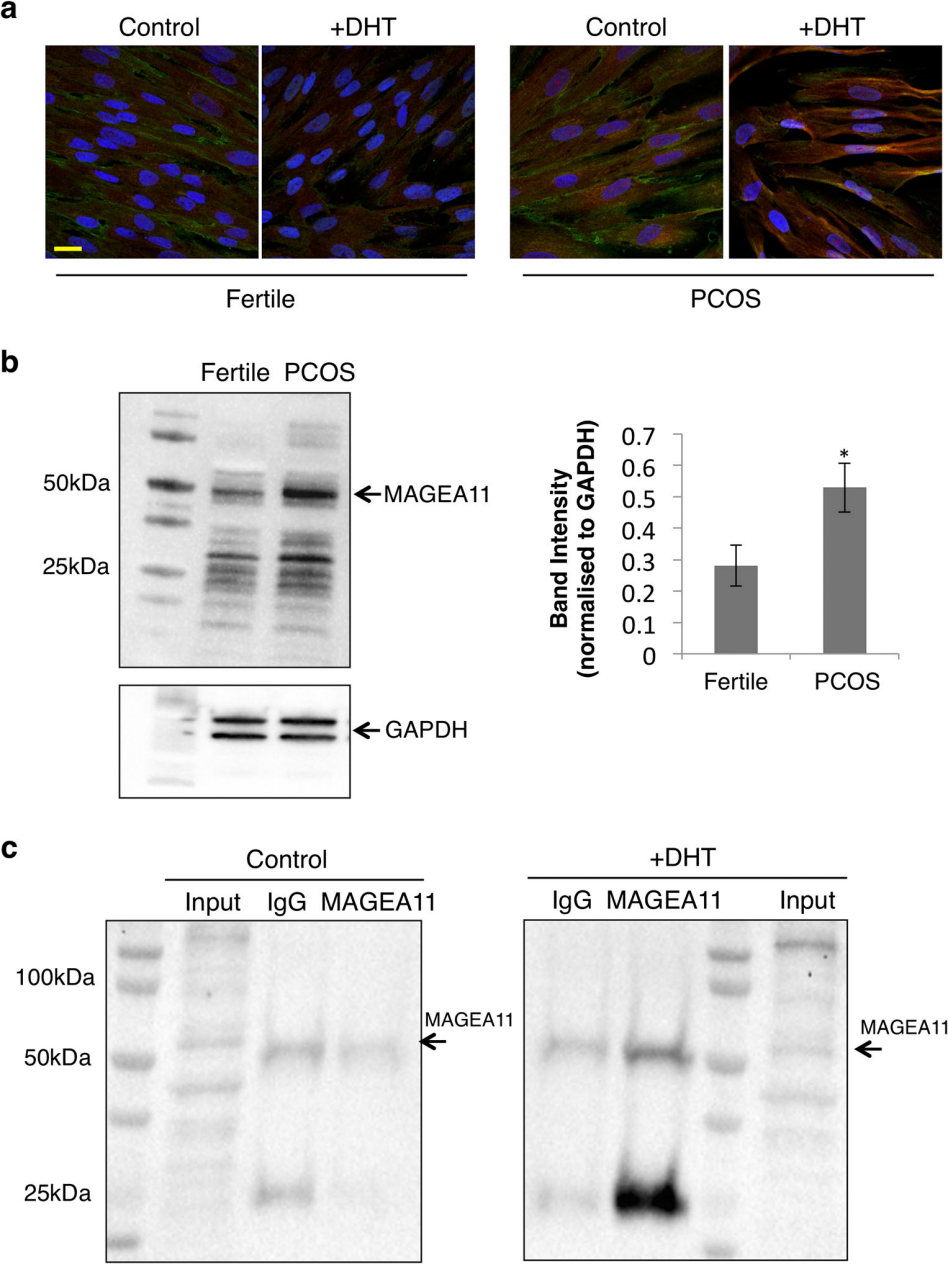
MAGEA11 protein localises to the nucleus of endometrial stromal cells and interacts with AR in PCOS patients. **a** Immunofluorescence confocal microscopy images of fertile and PCOS hESCs that were either left untreated (control, left panel) or treated with DHT for 48 h (DHT, right panel). ESCs were obtained from cells in the secretory phase of the cycle. Fixed cells were incubated with anti-AR and anti-MAGEA11 antibodies for detection. Scale = 20 μm. **b** Cell lysates from fertile (*n* = 3) and PCOS (*n* = 3) ESCs were subjected to western blot analysis. The left panel shows a representative blot of MAGEA11 protein expression and the right panel displays the corresponding densitometry; values were normalised to GAPDH expression and are expressed as average and standard deviation (SD). The statistical analysis was performed using Student’s *t* test; *p ≤ 0.05 is considered significant. **c** Cell lysates from PCOS ESCs were either left untreated (control, left panel) or treated with DHT for 24 h (right panel) and subjected to protein co-immuneprecipitation using an anti-AR antibody (N-20). Input was used as positive control and IgG as a negative control

Detailed analysis of AR and MAGEA11 distribution in PCOS samples revealed that they accumulate at high levels in both cytoplasmic and nuclear compartments (Figs. 2 and 3), whereas in cells from fertile patients, only low levels of AR and MAGEA11 staining were seen, and MAGEA11 was restricted to the cytoplasm. PCOS-specific nuclear co-localisation was also observed in vitro by confocal immunofluorescence microscopy when hESCs isolated from PCOS patients were treated with DHT (Fig. 4a and Supplementary Fig. 3). It appears that the two proteins could interact prior to nuclear importation, as per observed perinuclear or membrane-associated co-localisation (Fig. 4a and Supplementary Fig. 3).

Having demonstrated the temporal and spatial co-location of the two proteins, their direct molecular interaction was assessed, as such an interaction would support the notion that MAGEA11 functions as an AR nuclear co-regulator in the endometrial stroma, similar to its function in prostate cancer cells. Fertile and PCOS primary stromal cells were grown to confluence, and total protein extracted. Protein blot analysis revealed a major 50-kDa MAGEA11 isoform, corresponding to the canonical isoform one of the protein, which was expressed in both cell types (Fig. 4b). The bands observed in the 20–37-kDa range could be a result of cross-reactivity with other MAGEA11 isoforms, as well as with a number of highly homologous MAGE proteins (e.g., MAGEA4, 34.7 kDa) [21]. Densitometry analysis demonstrated that canonical MAGEA11 expression was significantly greater in PCOS samples confirming IHC and cytometric analysis (Fig. 4b). Subsequently, the co-immunoprecipitation analysis was carried out in order to demonstrate any direct molecular interaction between MAGEA11 and AR. In PCOS stromal cells treated with 0.1 μM DHT full-length ~ 50 kDa isoform one, MAGEA11 was detected at levels significantly above background (IgG), whereas no increase was observed in untreated cells (Fig. 4c). A second band (~ 25 kDa) was detected as a result of co-immunoprecipitation (Fig. 4c), likely a product of the degradation of MAGEA11, which is known to have a short half-life [22]. These data demonstrate an association between native AR and MAGEA11 isolated from primary endometrial stromal cells, and given previous studies have shown that the MAGEA11-AR interaction has an absolute requirement for DHT, this suggests that such a complex would only form in PCOS patients with hyperandrogenaemia.

### Characterisation of AR targets in PCOS

**Fig. 5:**
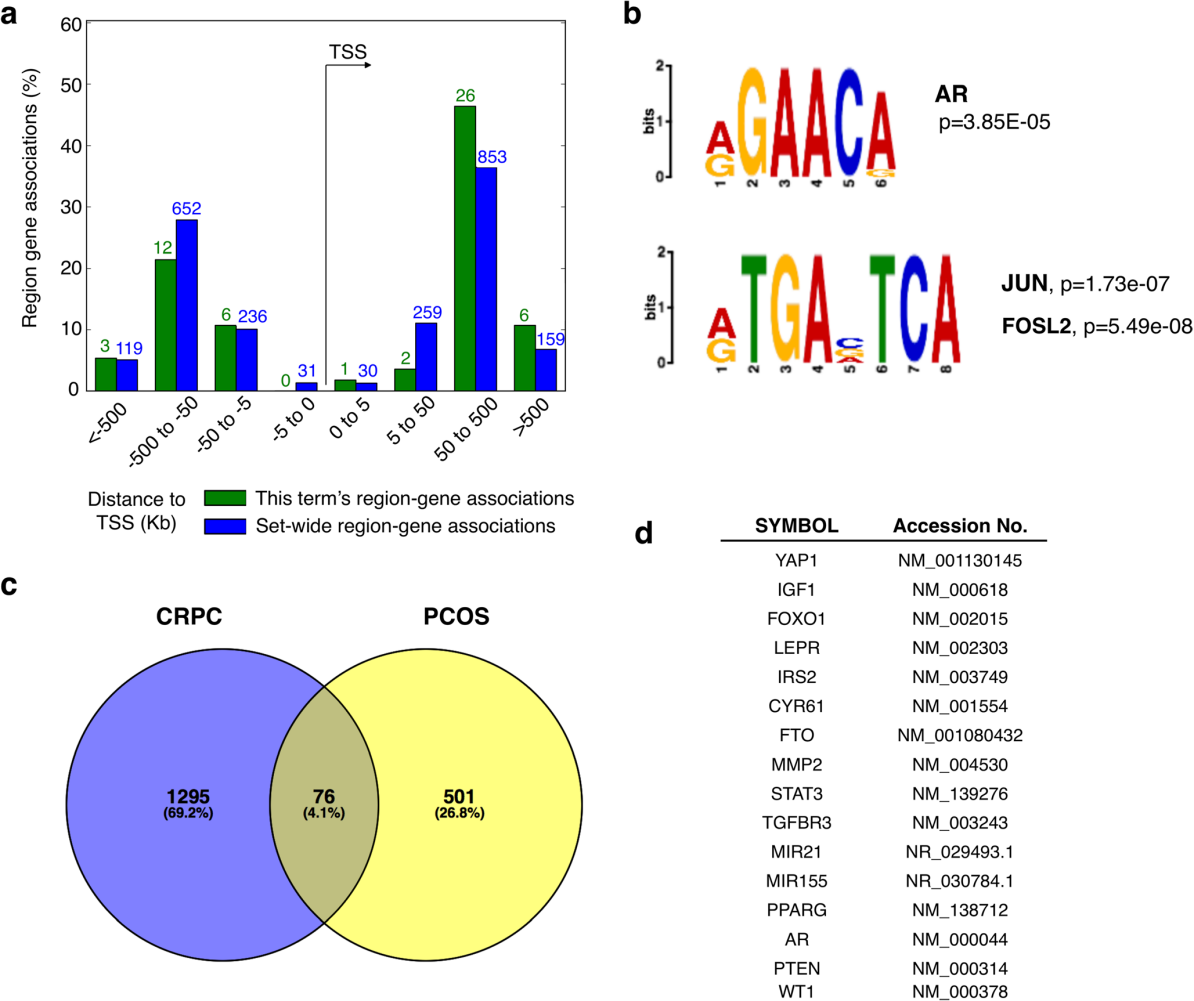
Genome-wide analysis of AR targeting in PCOS stromal cells. **a** Diagram showing the average distribution of AR binding sites surrounding gene transcription starting sites (TSSs) in human PCOS tissue. This diagram was generated using the Genomic Regions Enrichment of Annotations Tool (GREAT). Blue columns represent the whole set of filtered AR peaks and green columns represent AR peaks directly linked to the PCOS disease, according to GREAT databases. **b** Sequence logos and correspondent *p* values of transcription factor binding motifs enriched for AR in human PCOS tissue, generated with the MEME-Suite application. **c** Venn diagram showing the overlap between AR binding sites identified in human PCOS tissue and castrate-resistant prostate cancer (CRPC) [23]. **d** Table containing the list of genes identified as targets of AR in PCOS tissue, which are also involved in endometrial cancer disease, according to the NCBI databases

AR expression in PCOS stromal cells is likely to drive transcriptional re-programming and, as such, has a direct role in the delay of endometrial cell differentiation (Figs. 2 and 3). We therefore conducted genome-wide localisation experiments using chromatin immunoprecipitation coupled with DNA sequencing (ChIP-seq) to understand which transcriptional networks were likely to be regulated by AR. ChIP-seq chromatin isolated from DHT-treated PCOS hESCs probed with an anti-AR antibody identified 6296 loci (FDR 0.06%) that were enriched for AR, with no Y chromosome representation (Table 2; Supplementary Table 1). The majority of AR binding occurred distal to the transcription start site, at distances between − 500 and − 50 kb (Fig. 5a). To confirm the validity of the AR targets identified, we tested for the presence of consensus AR binding sites (R**AAC**R) using the MEME software [24] and demonstrated that the nuclear receptor specifically recognised its cognate binding sequence (*p* = 3.85E− 05) (Fig. 5b). In addition, AR also displayed highly specific recognition for JUN binding sites (*p* = 1.725E− 07, R**TGA**V**TCA**) and Sp1 (data not shown). JUN proteins are components of the AP-1 transcription activator family that have previously been shown to interact with, and enhance AR activity in prostate and prostate cancer cells [25, 26].

**Table 2:** Top androgen receptor targets in endometrial (PCOS) and castrate-resistant prostate cancer tissues

PCOS specific			Common PCOS-CRPC (prostate)		
Symbol	Peak value	Accession No.	Symbol	Peak value	Accession No.
DUSP27	130	NM_001080426	HFM1	185	NM_001017975
MIR663	104	NR_030386	SLC25A44	153	NM_014655
LOC100505875	100	XR_112656	PMF1	153	NM_007221
LOC100506842	94	XR_112632	PDE3A	114	NM_000921
NFXL1	94	NM_152995	NLE1	109	NM_001014445
MEF2D	87	NM_005920	UNC45B	109	NM_173167
C14orf145	84	NM_152446	YEATS2	105	NM_018023
TAF8	82	NM_138572	ABCC5	68	NM_005688
ZRANB2	74	NM_005455	DNAJB12	54	NM_001002762
PPM1D	74	NM_003620	LRRFIP2	47	NM_017724
TRIM56	74	NM_030961	PYGB	45	NM_002862
LTBP4	70	NM_003573	GTDC1	44	NM_024659
ENTPD4	69	NM_004901	BATF	40	NM_006399
TOP	64	XR_109290	C6orf81	40	NM_145028
MIR1826	64	NR_031727	FAM105A	39	NM_019018
MMP19	63	NM_002429	BLK	38	NM_001715
RANBP3	63	NM_007322	RGS22	36	NM_015668
C19orf45	63	NM_198534	ROCK1	35	NM_005406
ZNF358	63	NM_018083	SOX13	34	NM_005686
MCOLN1	63	NM_020533	KLF9	33	NM_001206

Gene ontology analysis revealed that AR targets were predominantly involved in processes related to the positive regulation of cell death, including apoptosis (Supplementary Table 2). This finding supports our early observations showing that apoptosis is delayed in PCOS, and suggests that the overexpression and hyperactivation of AR may be responsible for this. In addition, a number of specific AR targets including KLF9, KLF13, and PLZF were identified, all of which have roles in decidualisation [27, 28].

As AR functions predominately in male reproductive tissue, we asked whether the endometrial AR targets and regulatory pathways identified through our ChIP-seq analysis were tissue and gender-specific. Comparison of endometrial AR targets (577 genes) with AR targets from a prostate-specific data set (1371 genes) [23] revealed a set of targets that were specific to endometrial tissue (501 genes, Table 2), as well as a set of common AR targets (76 genes, Table 2). Common PCOS-prostate targets include a number of genes involved in well-known carcinogenic processes such as Rho-GTPase signalling (ROCK1) [29] or the NF-κβ pathways (PYGB) [30], mechanisms commonly associated with the regulation of apoptosis. These findings indicate that AR targets are predominantly gender specific, although there is an overlap that highlights the connection between male and female reproductive malignancies.

Finally, as PCOS patients have an increased risk of developing endometrial cancer (EC), we considered whether any AR targets were implicated in this disease. Comparison of our data with a list of EC-related genes obtained from PubMed databases revealed a set of known oncogenic targets (16 genes, Fig. 5d), including tumour suppressor PTEN, whose expression is commonly lost in endometrial cancer tissues [31], the matrix metalloproteinase-2 (MMP2) and YAP1, which are commonly found overexpressed in endometrial cancers [32, 33]. Additionally, our comparisons identified the tumour suppressor gene WT1, whose expression is downregulated in PCOS endometrium during implantation [4].

## Discussion

The window of implantation during the female reproductive cycle is a tightly controlled process, which if perturbed can result in the inability of the endometrium to receive and implant a blastocyst, and subsequently to establish the earliest phase of pregnancy. PCOS is a common multi-factorial disorder linked to female infertility. In women with PCOS symptoms who continue to ovulate, loss of normal endometrial function appears to be an underlying factor associated with infertility. Incorrect decidualisation, including timing and incomplete cellular transformation, can result in a non-receptive endometrium and incorrect intercellular signalling between the maternal tissue and the embryo.

A common feature of PCOS is elevated levels of circulating androgens—hyperandrogenaemia. This coincides with increased and temporally abnormal expression of the AR in endometrial tissue, which could lead to the activation of AR-driven transcriptional programmes. AR is a DNA-binding transcription factor, the function of which is to recruit co-regulator proteins to target genes resulting in either the activation or repression of the transcription of these targets. MAGEA11 has been described as a co-activator of AR in prostate tissues. Here, we show that the expression of MAGEA11 is increased in the endometrial stroma of PCOS tissue and cells isolated from this stromal compartment treated with DHT. This is a PCOS-specific effect, as MAGEA11 does not increase in the stromal component of endometrial tissue from fertile controls, nor does DHT treatment of cells derived from this tissue result in increased MAGEA11 expression. MAGEA11 protein was present as the canonical 50 kDa isoform one in PCOS stromal cells, where it interacted directly with AR in the presence of DHT. This is the first time this AR-MAGEA11 interaction has been demonstrated with native proteins isolated from primary endometrial cells and verifies that the AR-MAGEA11 interaction previously shown using overexpressed proteins isolated from mammalian expression systems is indeed physiologically relevant.

AR chromatin immunoprecipitation experiments using PCOS stromal cells stimulated with DHT identified the direct targets of the nuclear hormone receptor. Krüppel-like factor (KLF)-9 and 13 transcription factors (KLF9/KLF13) were both major AR targets as determined by the depth of read. KLF9 is expressed in endometrial stromal cells but controls the response of adjacent luminal epithelial cells to progesterone [27]. In murine models, loss of *klf9* results in less post-implantation embryos, which appears to be due to increased levels of BMP2 in these animals. In hESC treated with cAMP, estradiol-17β (E2) and medroxyprogesterone acetate to induce decidualisation, as well as siRNA to knock down KLF9 expression, both KLF13 and BMP2 mRNA levels increase. Conversely, in the same decidualised hESC model, siRNA to knock down KLF13 results in the increase of KLF9 expression and reduction of BMP2. This KLF-BMP appears important as the endometrial stromal cells of *bmp2-*deleted mice are infertile [27]. Disruption of this signalling axis by AR binding to KLF9 and 13 could therefore be a cause of delayed/failed decidualisation in PCOS patients. The promyelocytic leukaemia zinc finger transcription factor (PLZF) was a second transcription factor identified as a direct AR target. PLZF appears to have a role in P4-dependant hESC decidualisation as it is normally induced by P4 and contains PR regulator regions, and controls EGR1 expression levels [28]. KLF9/13 and PLZF normally function through the PR transcription pathway. It appears overexpression and activation of AR could disrupt this normal high-level transcriptional programming via driving misexpression of these key transcriptional regulators in PCOS patients.

Interestingly, as well as binding to AR response elements, the nuclear receptor is also recruited to JUN response element-containing regulatory regions, presumably by a JUN response element-binding protein such as c-Jun, which interacts directly with AR in prostate cancer cells and enhances the trans-activation function of AR [25, 26].

Gene ontology analysis of AR chromatin immunoprecipitation data revealed that AR interacts with genes involved in the regulation of cell death and apoptosis including EGFR and WT1, the expression of which is reduced in PCOS [4], suggesting AR may act as a repressor at the WT1 locus. The consequences of this repression have been highlighted before [4] and substantiate a functional role of AR in decidualisation. Bcl2, a known WT1 target, becomes upregulated in PCOS patients following AR activation, delaying the cell cycle and inhibiting apoptosis via p27 [34]. β-catenin, repressed by WT1 in fertile cells [35], is expressed in PCOS women, and likely contributes to the abnormal upregulation of the Wnt pathway [4, 36]. The impact of AR targeting these genes appears to be manifested in the delayed and incomplete morphological transition of stromal cells isolated from PCOS endometrium where cells retain a more elongated mesenchymal-like phenotype and produce a limited amount of prolactin when treated with the decidualisation stimulus of cAMP.

Together, these data support the notion that elevated androgens result in delayed decidualisation and implicate AR and its co-regulator MAGEA11 in incorrect endometrial differentiation, the critical timing process of embryo implantation, and thus human reproduction.

## Electronic supplementary material

ESM 1 (DOCX 4642 kb)

ESM 2 (XLSX 819 kb)

ESM 3 (XLSX 11 kb)
