# Supplementary material for: Delayed endometrial decidualisation in polycystic ovary syndrome; the role of AR-MAGEA11
Source: J Mol Med (Berl). 2019 Jun 29;97(9):1315–27. doi: 10.1007/s00109-019-01809-6 (PMC6713698; doi:10.1007/s00109-019-01809-6)
Supplement: Supplementary file 1 — (DOCX 4642 kb) [file 109_2019_1809_MOESM1_ESM.docx]

**Supplementary Material**

**Delayed endometrial decidualisation in polycystic ovary syndrome; the role of AR-MAGEA11**.

Kinza Younas, Marcos Quintela, Samantha Thomas, Jetzabel Garcia, Lauren Blake, Helen Whiteland, Adnan Bunkheila, Lewis Francis, Lavinia Margarit, Deyarina Gonzalez, R. Steven Conlan.

**Supplementary Tables**

Supplementary Tables are included as separate excel files.

Supplementary Table 1 encompasses 6,296 loci identified after enriching DHT-treated PCOS hESCs with an anti-AR antibody.

Supplementary Table 2 encompasses the list of processes that involve predominant AR targets (peak value >20), according to gene ontology analysis software.

**Supplementary Figures**

**Supplementary Fig. 1 Morphological changes in ESC cells during *in vitro* decidualization**

**
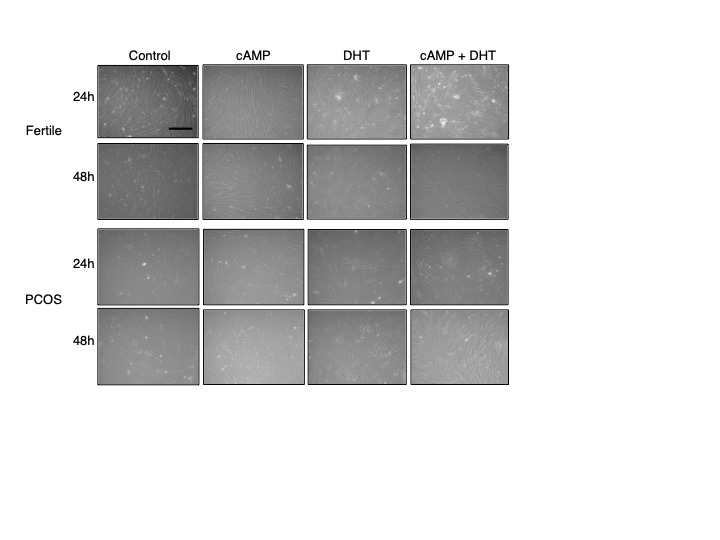
**

**Supplementary Fig. 2 Magnified MAGEA11 expression in proliferative/secretory endometrium**

**
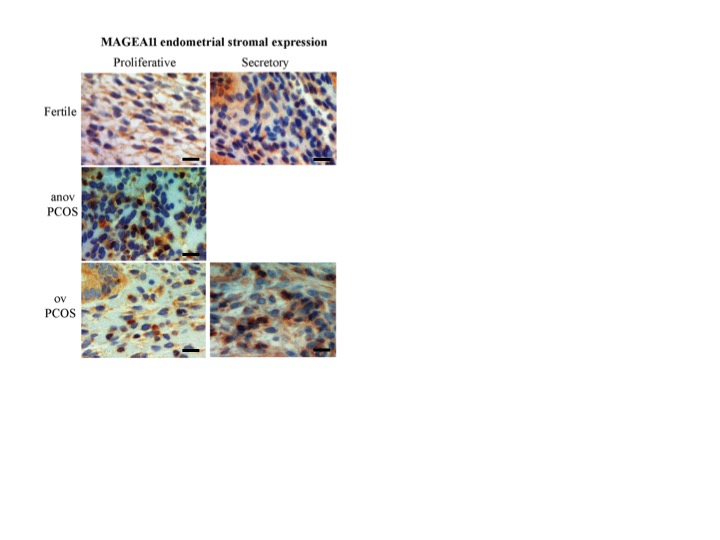
**

**Supplementary Fig. 3 Co-localization of AR and MAGEA11 proteins**


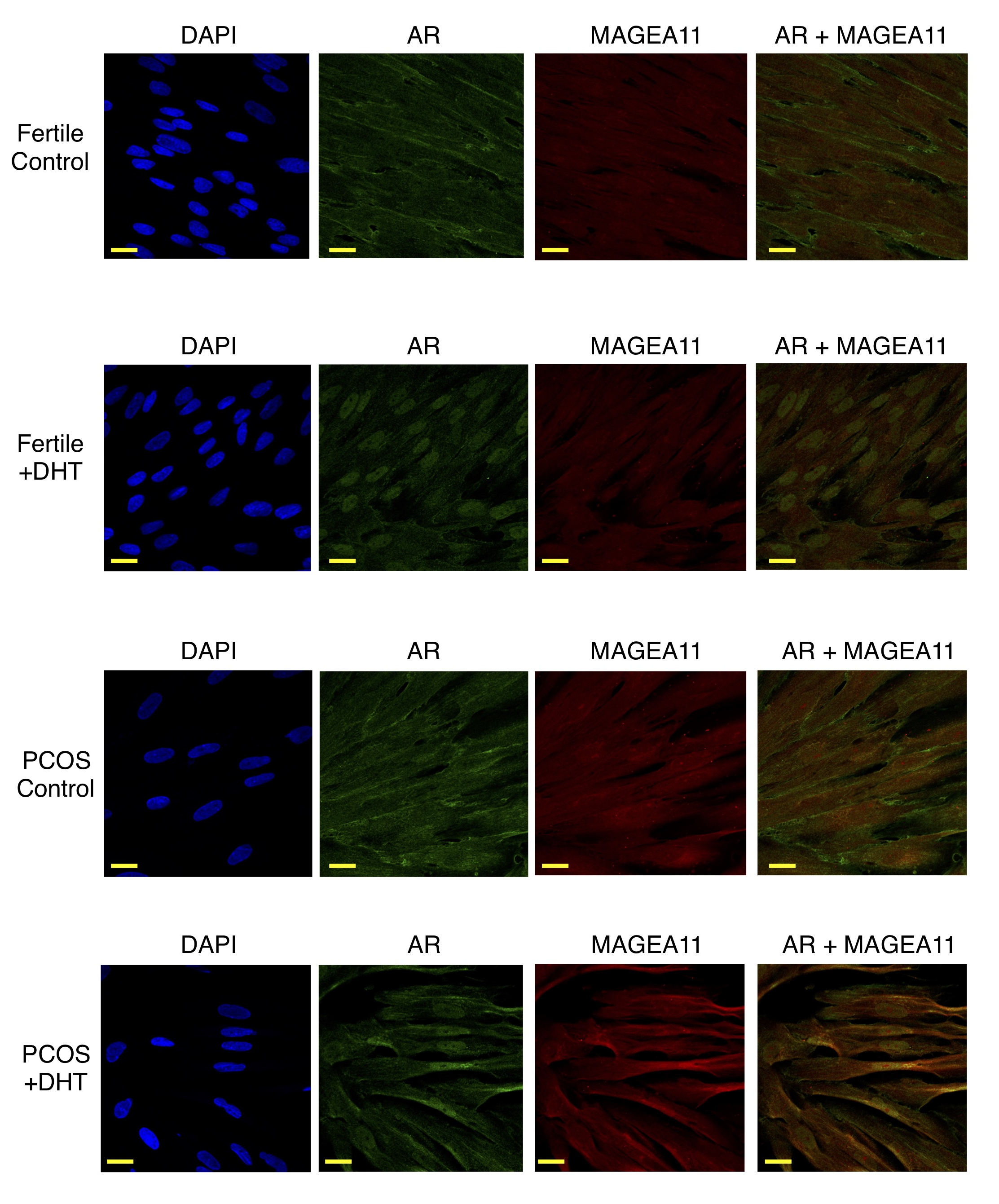


**Supplementary Figure Legends**

**Supplementary Fig. 1 Morphological changes in ESC cells during *in vitro* decidualization**

Representative images of endometrial stromal cell (ESC) shape changes during *in vitro* decidualization. ESCs were isolated from biopsies of fertile (n=8) and PCOS (n=9) patients and were either left untreated (C), incubated with cyclic AMP (cAMP, 0.5mM), dihydrotestosterone (DHT, 10^-6^M) or a combination of both for 24h and 48h. ESCs were obtained from cells in the secretory phase of the cycle. Scale = 40μm.

**Supplementary Fig. 2 MAGEA11 expression in proliferative/secretory endometrium**

Proliferative/secretory phase endometrium from fertile, anovulatory PCOS and ovulatory PCOS patients was analysed for the expression of MAGEA11 as described in Materials and Methods. Immunohistochemistry (IHC) images display x40 magnification. Scale = 20μm.

**Supplementary Fig. 3 Co-localization of AR and MAGEA11 proteins**

Immunofluorescence confocal microscopy images of fertile and PCOS hESCs that were either left untreated (Control) or treated with DHT for 48h (DHT). Fixed cells were stained with DAPI (blue, first column), anti-AR (green, second column) and anti-MAGEA11 (red, third column) for detection. The forth column represents a superposition of anti-AR and anti-MAGEA11 images. Scale =20μm.
